# Author Correction: Aryl amino acetamides prevent *Plasmodium falciparum* ring development via targeting the lipid-transfer protein *Pf*START1

**DOI:** 10.1038/s41467-024-52849-7

**Published:** 2024-09-30

**Authors:** Madeline G. Dans, Coralie Boulet, Gabrielle M. Watson, William Nguyen, Jerzy M. Dziekan, Cindy Evelyn, Kitsanapong Reaksudsan, Somya Mehra, Zahra Razook, Niall D. Geoghegan, Michael J. Mlodzianoski, Christopher Dean Goodman, Dawson B. Ling, Thorey K. Jonsdottir, Joshua Tong, Mufuliat Toyin Famodimu, Mojca Kristan, Harry Pollard, Lindsay B. Stewart, Luke Brandner-Garrod, Colin J. Sutherland, Michael J. Delves, Geoffrey I. McFadden, Alyssa E. Barry, Brendan S. Crabb, Tania F. de Koning-Ward, Kelly L. Rogers, Alan F. Cowman, Wai-Hong Tham, Brad E. Sleebs, Paul R. Gilson

**Affiliations:** 1 Burnet Institute, Melbourne, VIC 3004 Australia; 2 Walter and Eliza Hall Institute, Parkville, VIC 3052 Australia; 3 Institute of Mental and Physical Health and Clinical Translation (IMPACT) and School of Medicine, Deakin University, Geelong, VIC 3220 Australia; 4 Department of Medical Biology, The University of Melbourne, Parkville, VIC 3010 Australia; 5 School of Biosciences, The University of Melbourne, Parkville, VIC 3010 Australia; 6 Department of Microbiology and Immunology, The University of Melbourne, Parkville, VIC 3010 Australia; 7 Department of Molecular Biology, Umeå University, Umeå, 901 87 Sweden; 8 The Laboratory for Molecular Infection Medicine Sweden (MIMS), Umeå, Sweden; 9 Department of Infection Biology, Faculty of Infectious Diseases, London School of Hygiene and Tropical Medicine, WC1E 7HT London, UK; 10 Wellcome Trust Human Malaria Transmission Facility, Faculty of Infectious & Tropical Diseases, London School of Hygiene & Tropical Medicine, London, WC1E 7HT UK; 11 Monash University, 3800 Melbourne, VIC Australia; 12 Present Address: Department of Microbiology and Molecular Medicine, University of Geneva, Geneva, 1206 Switzerland

**Keywords:** Antiparasitic agents, Parasite biology, Malaria

Correction to: *Nature Communications* 10.1038/s41467-024-49491-8, published online 18 June 2024

The original version of the Supplementary Information associated with this Article contained an error in Supplementary Fig. 7c and d, in which the concentrations of W-991 were provided on the x-axes in the wrong order. The HTML has been updated to include a corrected version of the Supplementary Information.

