# Supplementary material for: Aryl amino acetamides prevent Plasmodium falciparum ring development via targeting the lipid-transfer protein PfSTART1
Source: Nat Commun. 2024 Jun 18;15:5219. doi: 10.1038/s41467-024-49491-8 (PMC11189555; doi:10.1038/s41467-024-49491-8)
Supplement: Supplementary file 1 — Supplementary Information [file 41467_2024_49491_MOESM1_ESM.pdf]

Aryl amino acetamides prevent *Plasmodium falciparum* ring development via targeting the lipid-transfer protein *Pf*START1

Madeline G. Dans[1,2,3,4#,*], Coralie Boulet[1,12#], Gabrielle M. Watson[2,4], William Nguyen[2,4], Jerzy M. Dziekan[2,4], Cindy Evelyn[2,4], Kitsanapong Reaksudsan[2,4], Somya Mehra[1,3], Zahra Razook[1,3], Niall D. Geoghegan[2,4], Michael J. Mlodzianoski[2,4], Christopher Dean Goodman[5], Dawson B. Ling[1], Thorey K. Jonsdottir[1,6,7,8], Joshua Tong[2], Mufuliat Toyin Famodimu[9], Mojca Kristan[10], Harry Pollard[10], Lindsay B. Stewart[10], Luke Brandner-Garrod[10], Colin J Sutherland[9, 10], Michael J. Delves[9], Geoffrey I. McFadden[5], Alyssa E. Barry[1,3], Brendan S. Crabb[1,6,11], Tania F. de Koning-Ward[3], Kelly L. Rogers[2,4], Alan F. Cowman[2,4], Wai-Hong Tham[2,4], Brad E. Sleebs[2,4] and Paul R. Gilson[1,6*]

1. Burnet Institute, Melbourne, Victoria 3004, Australia
2. Walter and Eliza Hall Institute, Parkville, Victoria 3052, Australia
3. Institute of Mental and Physical Health and Clinical Translation (IMPACT) and School of Medicine, Deakin University, Geelong, Victoria 3220, Australia
4. Department of Medical Biology, The University of Melbourne, Parkville, Victoria 3010, Australia
5. School of Biosciences, The University of Melbourne, Parkville, Victoria 3010, Australia
6. Department of Microbiology and Immunology, The University of Melbourne, Parkville, Victoria 3010, Australia
7. Department of Molecular Biology, Umeå University, Umeå 901 87, Sweden
8. The Laboratory for Molecular Infection Medicine Sweden (MIMS), Umeå, Sweden
9. Department of Infection Biology, Faculty of Infectious Diseases, London School of Hygiene and Tropical Medicine, London, WC1E 7HT, United Kingdom
10. Wellcome Trust Human Malaria Transmission Facility, Faculty of Infectious & Tropical Diseases, London School of Hygiene & Tropical Medicine, London, WC1E 7HT, United Kingdom
11. Monash University, Melbourne, Victoria 3800, Australia

12. Present affiliation: Department of Microbiology and Molecular Medicine, University of Geneva, Geneva 1206, Switzerland

#These authors contributed equally

*Correspondence and requests for materials should be addressed to: dans.m@wehi.edu.au, paul.gilson@burnet.edu.au

SUPPLEMENTARY INFORMATION

Supplementary Figures

Supplementary Tables

Supplementary Methods

Supplementary References

Supplementary Figures

A.

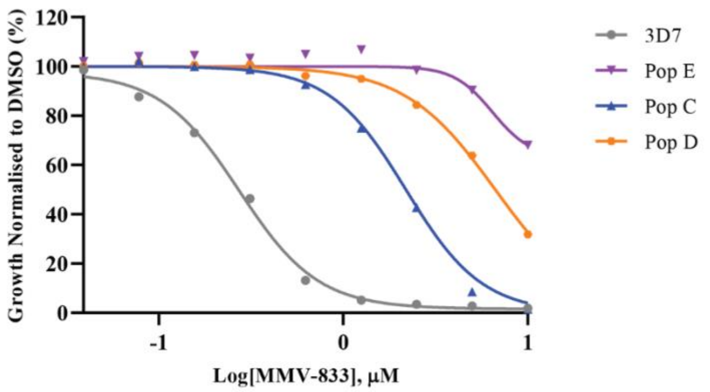

M-833 EC_{50} (μM)

3D7	PopE	PopC	PopD
0.27	>10	2.18	6.67

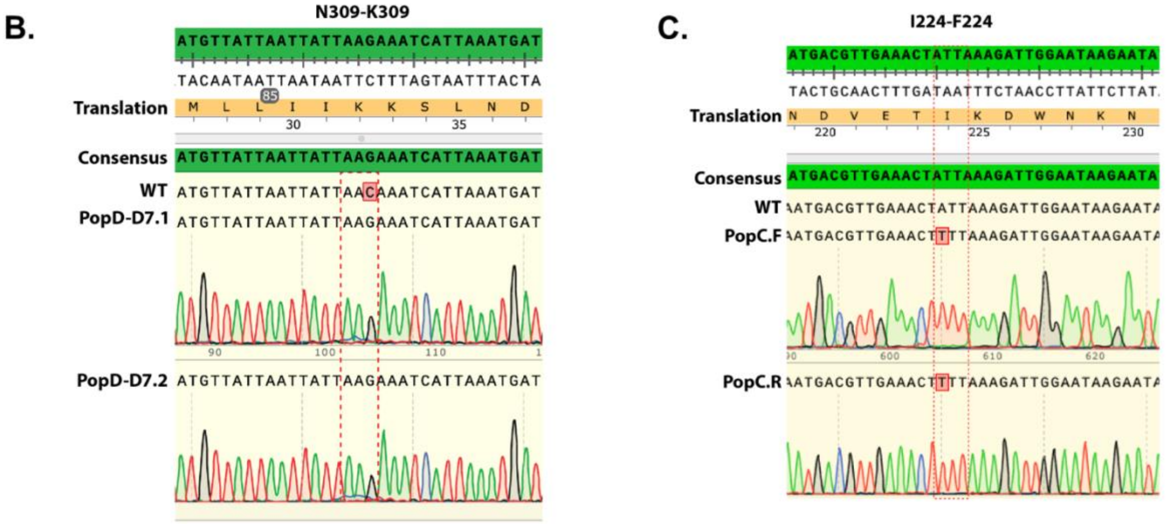

Supplementary Figure 1. Growth inhibition of M-833 resistant populations, *Pf*START1 mutations in PopD-D7 and PopC. (A) Resistance against M-833 could be generated against three out of five populations of parasites eliciting an 8 to 37 fold increase in EC_{50}. Data points indicate average of two technical replicates. **(B)** *Pfstart1* was PCR amplified from gDNA of the M-833 resistant clone D7 from population D (PopD-D7), and its sequencing indicates that PopD-D7 also contains mutation N309K, similar to other clones from this resistant population. **(C)** Similarly, *pfstart1* from PopC

was PCR amplified and sequenced. PopC displayed a I224F mutation. Source data are provided as a Source Data file.

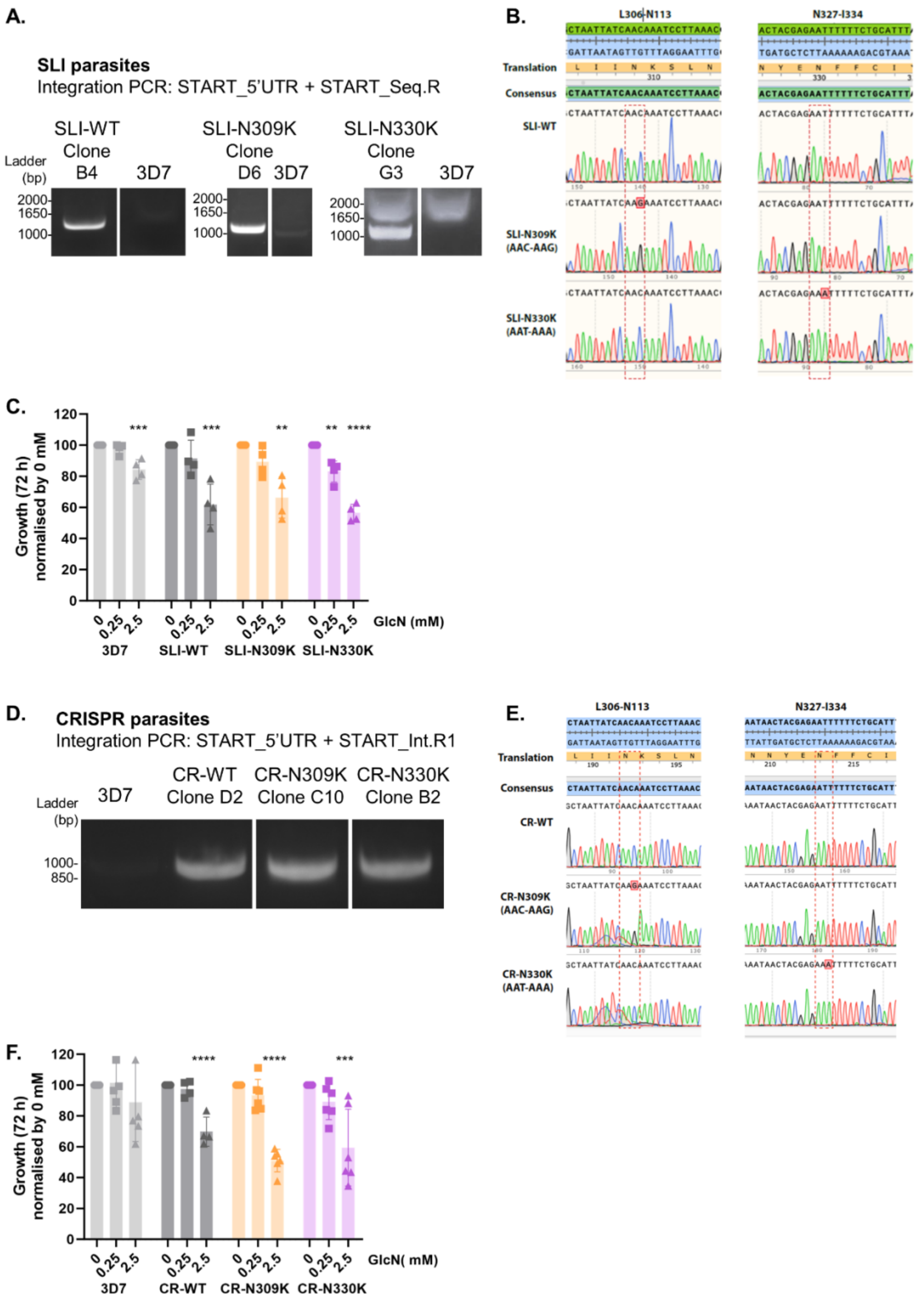

Supplementary Figure 2. Engineering *Pf*START1 WT, N309K or N330K using Selection Linked Integration (SLI) or CRISPR-Cas9 (CR). (A) PCR genotyping of SLI clones (SLI-WT clone B4, SLI-N309K clone D6 and SLI-N330K clone G3,

thereafter called SLI-WT, SLI-N309K and SLI-N330K). gDNA was extracted and a PCR was conducted to check for correct integration using primers START_5'UTR.F and START_Seq.R (Table S2); the same PCR on 3D7 gDNA was used as a control, as no bands should be observed in the 3D7 samples. **(B)** The sequencing of the PCR amplified *pfstart1* genes shows that SLI-WT has the wild-type version of *Pf*START1, and that SLI-N309K and SLI-N330K contain the correct mutations. **(C)** Growth of 3D7, SLI-WT, -N309K or -N330K over 72 h in the presence of 0, 0.25 or 2.5 mM GlcN was measured with an LDH assay. Growth was normalised to the 0 mM GlcN condition. n=4 biological replicates. Ordinary one-way ANOVA with Dunnett's multiple comparison test (comparison to the 0 mM condition). **: $p<0.005$. ***: $p<0.0005$. ****: $p<0.0001$. **(D)** Similar to SLI parasites, CRISPR transfectant parasites were cloned: CR-WT clone D2, CR-N309K clone C10 and CR-N330K clone B2, thereafter called CR-WT, CR-N309K and CR-N330K. A PCR was conducted to check for correct integration using primers START_5'UTR.F and START_IntR1; 3D7 gDNA was used as a control. **(E)** The PCR product was sequenced and indicated that CR-WT has the wild-type version of *pfstart1*, and that CR-N309K and CR-N330K contain the correct mutations. **(F)** Growth of 3D7, CR-WT, -N309K or -N330K over 72 h in the presence of 0, 0.25 or 2.5 mM GlcN was measured by LDH assay (same as **(C)**; n=6 biological replicates). Source data are provided as a Source Data file.

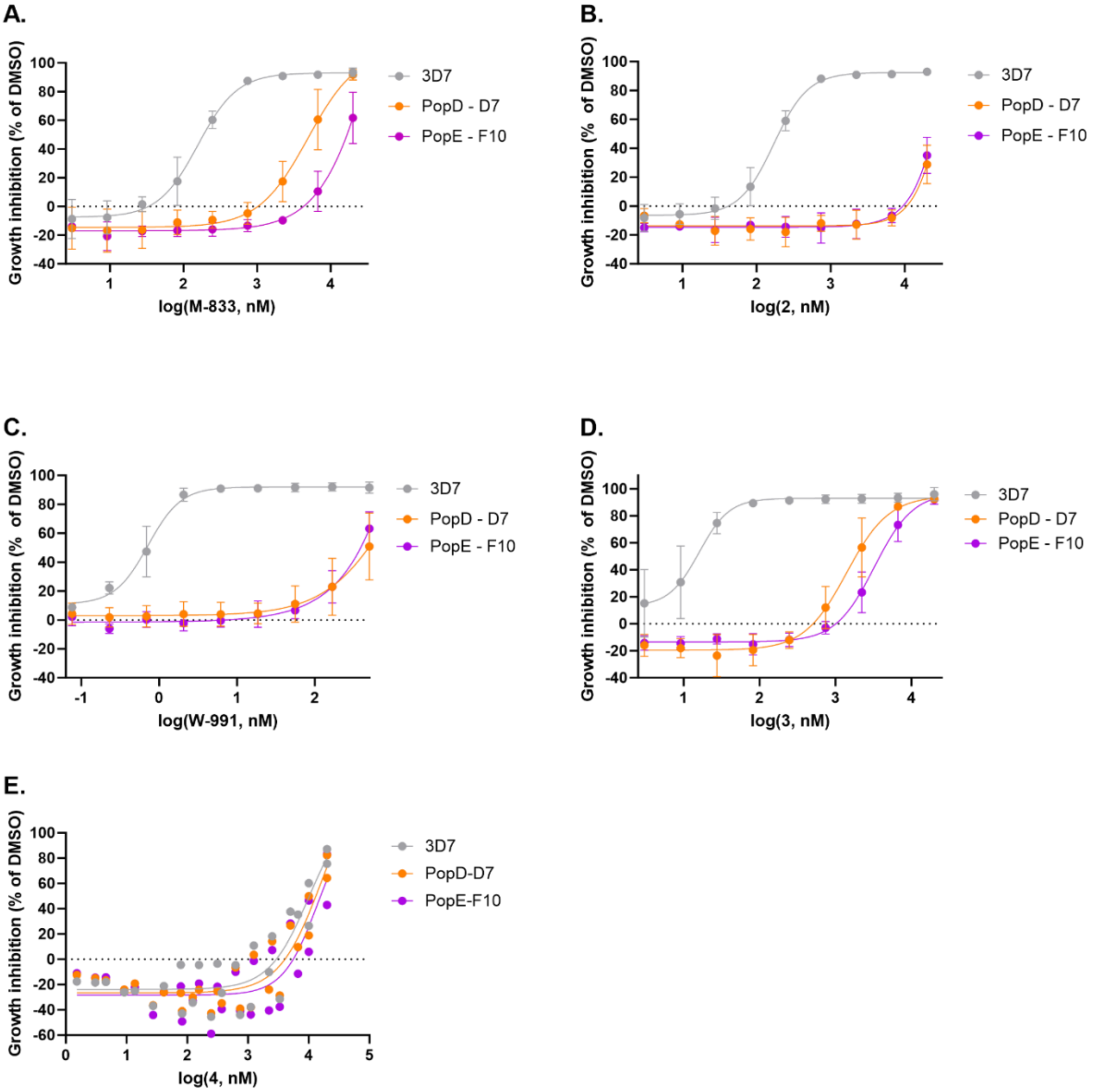

Supplementary Figure 3. Growth inhibition assays of M-833 and analogues on M-833 resistant parasites. Ring stage drug sensitive 3D7 parasites and M-833-resistant PopD-D7 (N309K mutant), and PopE-F10 (N330K mutant) parasites were exposed 72 h to a dilution series of M-833 **(A)**, compound **2 (B)**, W-991 **(C)**, compound **3 (D)**, and compound **4 (E)**. The structure of the compounds and the EC_{50} values can be found in Fig 4 and Table S1. N=3 biological replicates (mean +/- SD, except for **(E)** in which each point is an individual value). Source data are provided as a Source Data file.

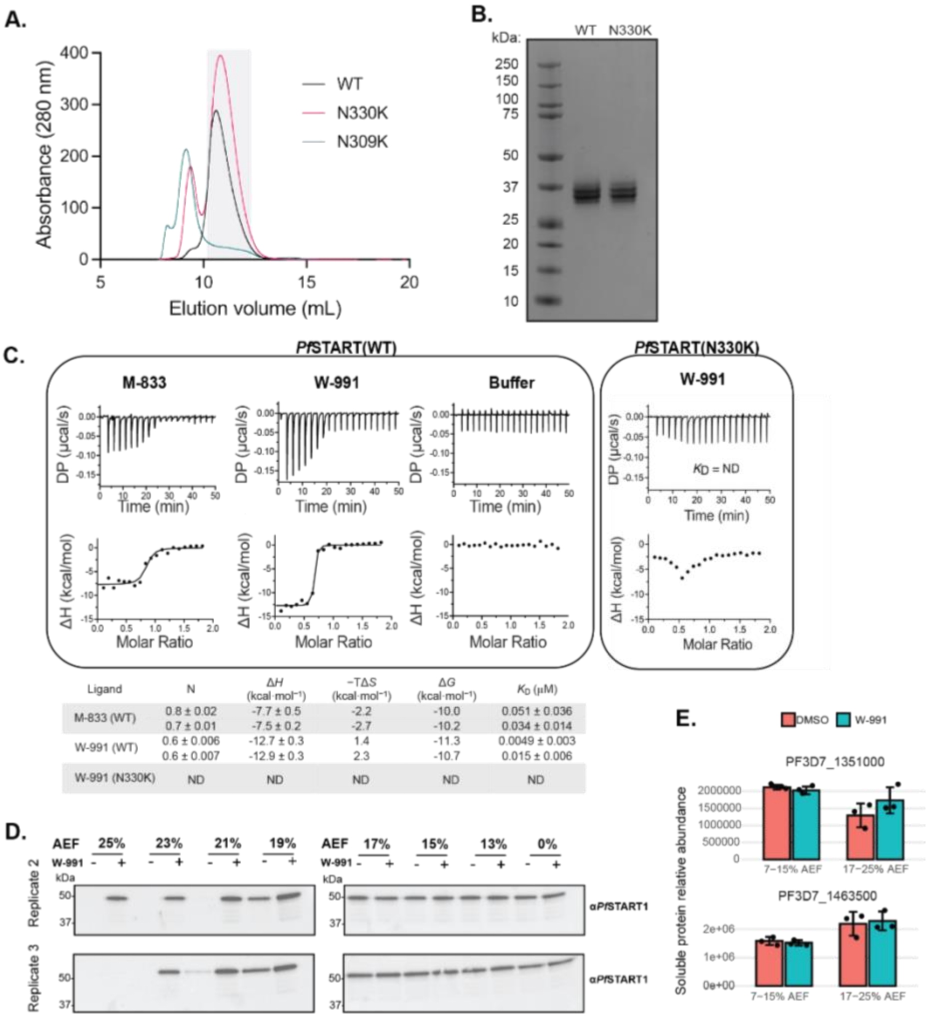

Ligand	N	ΔH (kcal·mol⁻¹)	−TΔS (kcal·mol⁻¹)	ΔG (kcal·mol⁻¹)	K_D (µM)
M-833 (WT)	0.8 ± 0.02	-7.7 ± 0.5	-2.2	-10.0	0.051 ± 0.036
	0.7 ± 0.01	-7.5 ± 0.2	-2.7	-10.2	0.034 ± 0.014
W-991 (WT)	0.6 ± 0.006	-12.7 ± 0.3	1.4	-11.3	0.0049 ± 0.003
	0.6 ± 0.007	-12.9 ± 0.3	2.3	-10.7	0.015 ± 0.006
W-991 (N330K)	ND	ND	ND	ND	ND

Supplementary Figure 4. Isothermal titration calorimetry analysis of M-833 series and *Pf*START1 and other START-domain containing proteins in solvent proteome profiling assays. (A) Size-exclusion chromatogram of recombinant WT *Pf*START1 (black), N330K (pink), and N309K (teal). Grey box indicates fractions used for ITC experiments. **(B)** SDS-PAGE analysis of recombinant *Pf*START1 proteins used for ITC experiments. **(C)** Second replicate thermograms of the M-833 series binding to recombinant *Pf*START1(WT) or *Pf*START1(N330K). The bottom panel comprises the data after integration of the peaks and a fitted offset applied. The binding curve shows the fit to a single-site binding model. DP = differential power. Summary of the individual thermodynamic parameters obtained for n=2. Error represents the error of

the fit. **(D)** Replicate western blots of solvent proteome assays (Fig 5B). **(E)** No significant difference was found between DMSO or W-991 treated parasite lysate in relative soluble protein abundance of Pf3D7_1351000 or Pf3D7_1463500 after solvent-induced protein precipitation, plotted for Gradient 1 (7-15% AEF) and Gradient 2 (17-25% AEF). Statistical analysis performed via a moderated t-test based on limma package. N=3 biological replicates, mean +/- SD where error bars represent the standard deviation. Source data are provided as a Source Data file.

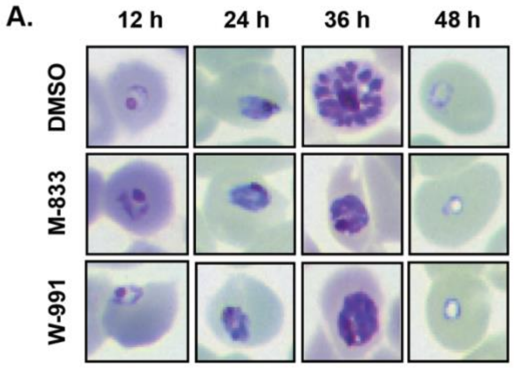

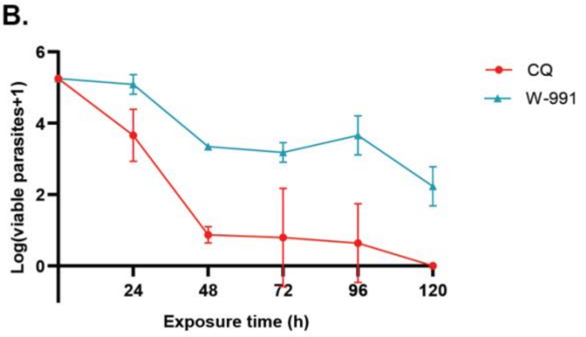

	CQ	W-991
Lag phase (h)	0.0	>24
Log(PRR)	3.9 (0.9)	1.3 (0.3)
99.9% PCT (h)	51.8 (12.9)	128.0 (12.8)

Supplementary Figure 5. *Pf*START1 inhibitors have no effect on ring or trophozoite development and exhibit a slow killing profile in a parasite reduction ratio assay. (A) Highly synchronous ring-stage 3D7 parasites were exposed to M-833 (2 µM), W-991 (60 nM) or DMSO (0.02%) and every 12 h for one growth cycle, Giemsa-stained thin blood smears were taken to visualise parasite morphology. **(B)** Ring-stage parasites were exposed to 10xEC_{50} of chloroquine (CQ) or W-991 for up to five days. An aliquot of the treated culture was removed daily, compounds were washed out and the parasites serially diluted. After 21 days, parasite growth was quantified via LDH assay and log(viable parasites+1) was calculated. W-991 demonstrated a ‘slow’ killing profile when compared to the fast-acting CQ. W-991 showed more than 24 h of lag phases (0-24 h, 48-96 h) whereby it did not reach its maximum rate of killing. Log parasite reduction ratio (log(PRR)) was calculated by the reduction of three log units between one cell cycle (0-48 h). Parasite clearance time (PCT) was calculated using a linear regression. Error bars represent the standard deviation of three biological replicates (values in brackets in table) and linear regressions were calculated using GraphPad Prism. Concentrations used equated to 75 nM and 60 nM for CQ and W-991, respectively. Source data are provided as a Source Data file.

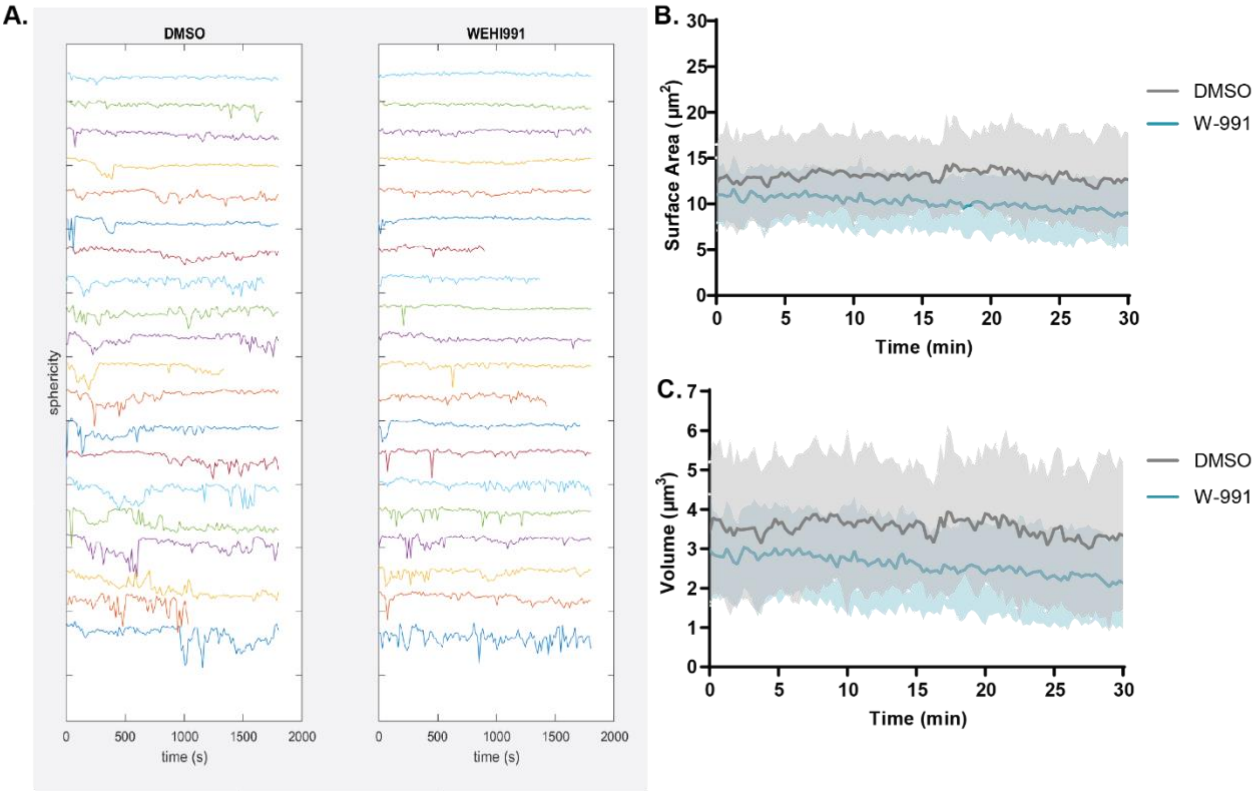

Supplementary Figure 6. Measurements of parasite vacuoles after merozoite invasion using lattice light sheet microscopy. (A) Individual vacuole sphericity quantification directly after merozoite invasion for both DMSO and W-991 treatments. Averages can be seen in Fig 7B. A reduction in both the mean surface area (+/-SD) **(B)** and volume (+/-SD) (**C)** of the vacuoles was also observed upon W-991 treatment which was found to not be significant via nested t-tests at 15 mins between DMSO and W-991-treated parasites (p=0.109 and p=0.129, respectively). Source data are provided as a Source Data file.

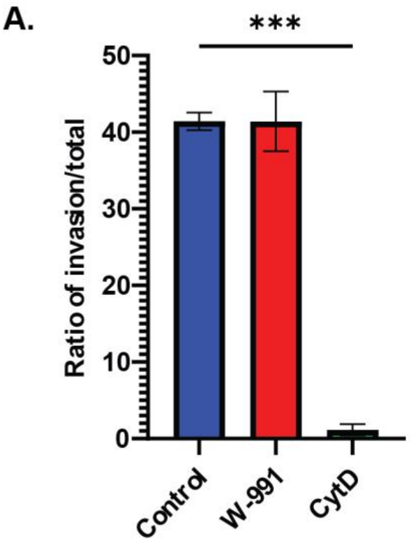

B.

Compound	Pf female gamete formation	Pf male gamete formation
M-833	-1.42%	0.87%
W-991	-2.93%	-1.33%

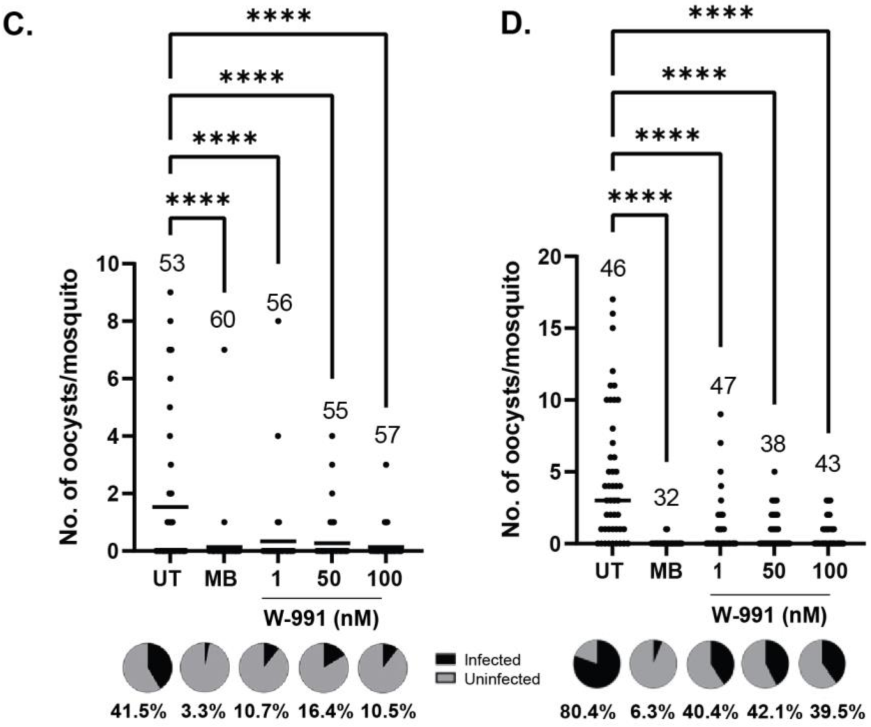

Supplementary Figure 7. W-991 shows no inhibition of sporozoite invasion or gamete formation but has transmission blocking activity. (A) *In vitro* HC-04 human liver cells were incubated with DMSO, W-991 or cytochalasin D (cytD) in the presence of 30,000 *P. berghei* sporozoites expressing mCherry for 2 h, followed by fixation with 4% paraformaldehyde. W-991 treatment showed no defect in invasion as measured by comparing mCherry (all sporozoites) and anti-*Pb*CSP antibody

fluorescence (uninvaded sporozoites) when compared with vehicle control. Statistical analysis performed via an Ordinary one-way ANOVA comparing DMSO and drug treatments. Error bars represent the standard deviation of two replicates. *** $P < 0.001$. **(B)** Values indicate percent inhibition of 1 µM M-833 and W-991 against female and male gamete formation in *P. falciparum*. **(C-D)** Replicates two and three of standard membrane feeding assays. UT indicates untreated and MB indicates methylene blue infectivity blocking control. Number of mosquitoes dissected is listed above each condition and infectivity rate is displayed as pie charts (bottom). Statistical analysis was performed via an Ordinary One-way ANOVA comparing UT to the other treatments. **** indicates p<0.0001. Source data are provided as a Source Data file.

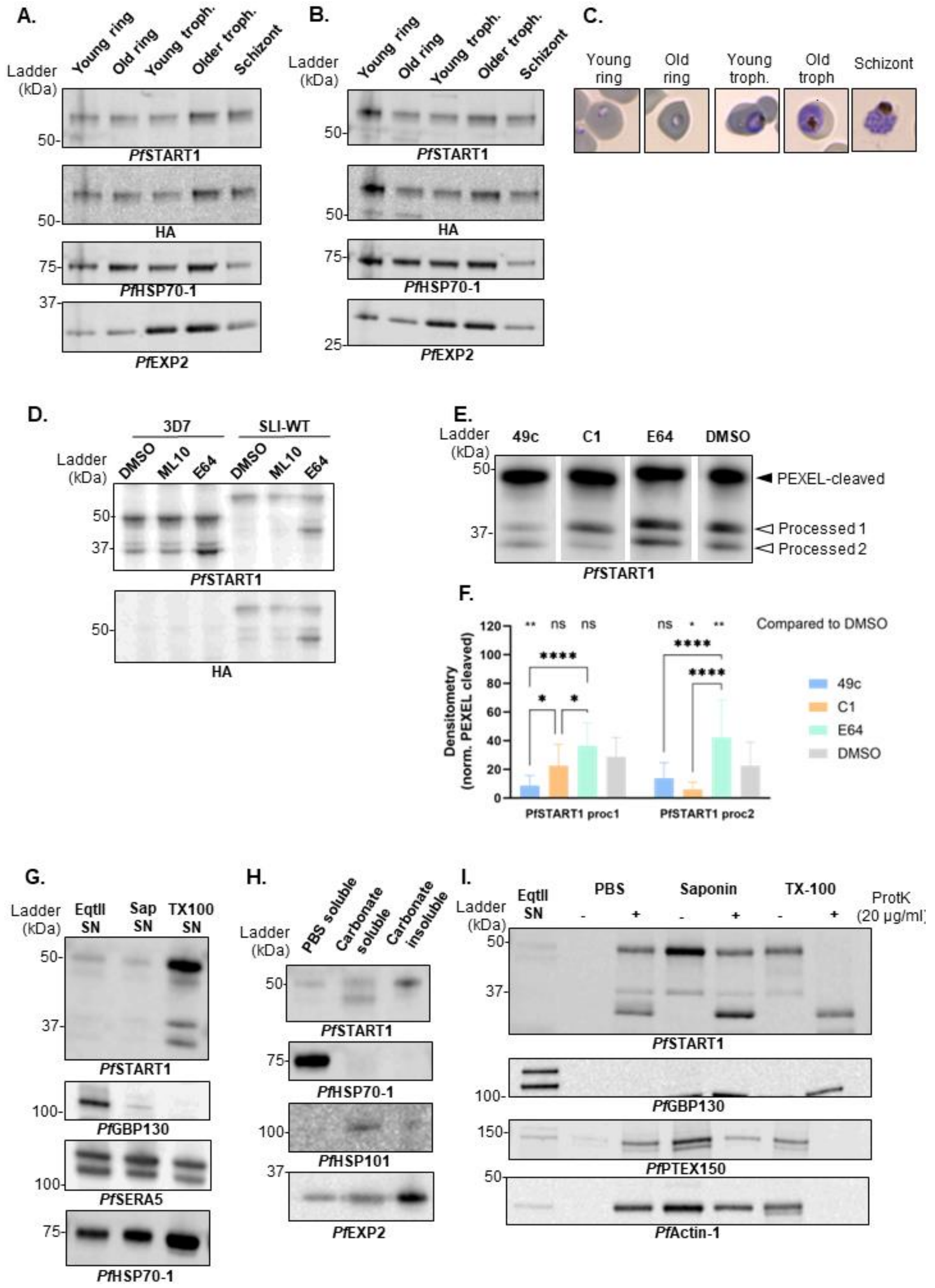

Supplementary Figure 8. *Pf*START1 expression, processing and localisation. (A-B) SLI-WT parasites were saponin-lysed at young- and old- ring, young- and older-trophozoite, and schizont stages (third replicate and densitometry are shown in Fig 8).

(C) Representative smears of corresponding parasites. **(D)** Synchronised 3D7 and SLI-WT schizonts were stalled at different stages of egress: 4 h with 25 nM ML10 (PKG inhibitor), 10 µM E64 (cysteine protease inhibitor) or allowed to progress (DMSO). Schizonts were saponin-lysed. **(E)** Synchronised 3D7 schizonts were magnet-purified, stalled at different stages of egress: 4 h with 10 nM 49c (plasmepsin X inhibitor), 1.5 µM compound 1 (C1; PKG inhibitor), 10 µM E64, or allowed to progress (DMSO). Whole cells were harvested. PEXEL-cleaved, and the two further processed forms of *Pf*START1 are indicated by solid and empty arrows respectively. **(F)** Densitometry of processed *Pf*START1 (proc1 and proc2) was normalised to the corresponding PEXEL-cleaved *Pf*START1. n=10 replicates (n=6 for the 49c treatment). Two-way ANOVA with Šídák's multiple comparisons test (compare each treatment to one-another). *: $p<0.05$. **: $p<0.005$. ***: $p<0.0005$. ****: $p<0.0001$. **(G)** To localise *Pf*START1, Percoll-purified 3D7 schizonts were sequentially lysed with equinatoxin II (EqtII), saponin (Sap) and Triton-X100 (TX100), and the supernatants (SN) were collected. *Pf*GBP130 is exported into the red blood cell (RBC) cytosol; *Pf*SERA5 localises in the parasitophorous vacuole (PV); *Pf*HSP70-1 is in the parasite cytosol. Another replicate is shown in Fig 8. **(H)** To determine the solubility of *Pf*START1, saponin-lysed 3D7 schizonts were sequentially lysed: the supernatant from the PBS lysis (freeze-thaw cycles, "PBS soluble"), the supernatant and the pellet from the sodium carbonate lysis ("Carbonate soluble" and "Carbonate insoluble" respectively) were collected. *Pf*HSP70-1 is soluble, *Pf*HSP101 is membrane-associated protein, and *Pf*EXP2 is a transmembrane protein. Another replicate is shown in Fig 8. **(I)** Proteinase K protection assay: Percoll-purified 3D7 schizonts were first lysed in EqtII. The remaining parasite and PV were incubated in PBS, saponin or TX-100, with or without protK. *Pf*GBP130 is a RBC cytosolic protein; *Pf*PTEX150 is a

PV protein; *Pf*Actin-1 is a parasite cytosolic protein. Another replicate is shown in Fig 8. Source data are provided as a Source Data file.

Supplementary Tables

		3D7	PopD – D7	PopE – F10
**M-833**	**EC_{50}**<br><br>**(CI)**	156 nM<br><br>(121.8 to 198.4)	4.88 µM<br><br>(2.889-128.972)	>20 µM<br><br>(N/A)
	Fold change compared to 3D7	-	x 31	> x 128
**2**	**EC_{50}**<br><br>**(CI)**	171 nM<br><br>(142.7-202.8)	>20 µM<br><br>(N/A)	>20 µM<br><br>(N/A)
	Fold change compared to 3D7	-	> x 117	> x 117
**W-991**	**EC_{50}**<br><br>**(CI)**	0.72 nM<br><br>(0.5361-0.8852)	>200 nM<br><br>(N/A)	>200 nM<br><br>(N/A)
	Fold change compared to 3D7	-	> x 278	> x 278
**3**	**EC_{50}**<br><br>**(CI)**	16 nM<br><br>(6.950-?)	1.4 µM<br><br>(1.016-2.019)	3.25 µM<br><br>(2.575-4.443)
	Fold change compared to 3D7	-	x 88	x 203
**4**	**EC_{50}**<br><br>**(CI)**	10.03 µM<br><br>(3.685-?)	15.73 µM<br><br>(5.059-?)	13.84 µM<br><br>(9.919-?)
	Fold change compared to 3D7	-	x 1.6	x 1.4

Supplementary Table 1. EC_{50} values of M-833 analogues on M-833-resistant clones. PopD-D7 contains the *Pf*START1 mutation N309K; PopE-F10 contains the *Pf*START1 mutation N330K. CI: 95% confidence interval. Fold-change compared to the EC_{50} on 3D7 parasites is also indicated.

Primer name	Sequence	Purpose
SLTP_1F	AGATCTTTGTCTTTTGTTTTCTCTGTTGTGGAGCT	Amplify *pfstart1* 5’ homology flank from 3D7 gDNA
SLTP_2R	CCAAGGAAGTCCATTAATAAGATATAAATATTTTCT	
SLTP_3F	ATATCTTATTAATGGACTTCCTTGGCCaTTcAAgAGCCAGGAC	Amplify recodonised *pfstart1* and add overlap with native 5’ flank and Pst cutting site
SLTP_PstR	CTGCAGcATCTTTATTGAAGAAGATACCGAATATTTTCTTGAAGAAGTTGA	
SLTP_EcoR1	GAATTCCCTTTTAAAAGTCAGGATACTATATATGAAGTGTATCA	Amplify *pfstart1* 3’ homology flank from 3D7 gDNA
SLTP_KasR	GGCGCCTTAGTCCTTATTAAAAAATATACCAAATATTTTTTTAAAAAAGTTAACGT	
START_5’UTR.F	TGTAATAATTTATACATTTTTATATCAGTTTATTTATTTTTGAGAAGGA	Amplify DNA only in parasites that have integrated the construct (used in SLI constructs)
START_Seq.R	ACTCATAACATATTTCACGCCTTTGTCATAAGAGT	
START_Int.R1	TCAAACACATCGTTTAAGGATTTGTTGATAATTAGCAGCA	With START_5’UTR.F: amplify DNA in parasites that have integrated the CRISPR construct
START_Int.R2	ACCATATGAGTTTTCTGGGTCAGTCTGCACGTGC	With START_3F: used to amplify the *pfstart1* locus to be sequenced in CRISPR parasites
Recodonised 3’ flank of START1:<br>• WT (N309 and N330)<br>• N309K mutant (underlined in brackets)<br>• N330K mutant (bolded, in brackets)	CCaTTcAAgAGCCAGGACACCATATACGAAGTGTACCAAAAATACTACAACAACAAGAATATGCTGCTAATTATC*AAC(AAG)*AAATCCTTAAACGATGTGTTTGATAATAATAGTAGTTATGCCAGGATAAATAACTACGAG***AAT(AAA)***TTTTTCTGCATTTACCCGAAATCAAAAAACTCTTACGACAAAGGTGTCAAGTACGTTATGAGCATCATATATGATGTGAATATCCCTAAGTTCATACAAAACAATATCCTAAACCAGATaTTCCCGGATCTTATATTCAATTTGCATAATACATCTATAGCTATAACTAATAAGACGGTGGGGACAGTGGTAGACTTATCCAAAAATGAACAGAATGCTTGGCACGCGCACAGTCTAAAAAACGTGAAACCAGAAGATACTCCAAATACTGAGCACGTGCAGACTGACCCAGAAAACTCATATGGTTTGGGGTTCATAAAGATGATATTTGTCGACGGTCCCTATAACCTGTGGATAATAAATGTCAACTTCTTCAAGAAAATATTCGGTATCTTCTTCAATAAAGAT	
gRNA	/AltR1/rArUrArGrUrArUrCrCrUrGrArCrUrU	Guides the recombinant Cas9 enzyme to cut the native *pfstart1* locus (but not the recodonised *pfstart1* DNA template)

Supplementary Table 2. List of primers used in this project.

**Antibody**	**Raised in:**	**Provider**	**Dilution** (WB: western blot; IFA: immunofluorescent assay)
HA	Mouse	Sigma	1:1000 (WB)
*Pf*START	Rabbit	WEHI (see Methods)	1:1000 (WB)
*Pf*EXP2	Mouse	WEHI (PMID: 19536257)	1:2000 (WB)
*Pf*EXP2	Rabbit	WEHI (PMID: 19536257)	1:2000 (WB)
*Pf*HSP70.1	Rabbit	WEHI (PMID: 28732045)	1:2000 (WB)
*Pf*GBP130	Mouse	WEHI	1:1000 (WB)
*Pf*SERA5	Rabbit	WEHI (PMID: 13679369)	1:1000 (WB)
*Pf*HSP101	Rabbit	WEHI (PMID: 19536257)	1:1000 (WB)
*Pf*Actin-1	Mouse	WEHI (PMID: 22389687)	1:500 (WB)
Rabbit-Alexa Fluor Plus 680	Goat	Invitrogen	1:10000 (WB)
Mouse-Alexa Fluor Plus 800	Goat	Invitrogen	1:10000 (WB)

Supplementary Table 3. List of antibodies used in this project.

Supplementary Methods

Molecular biology and transfection of *P. falciparum*: Selection Linked Integration method.

To introduce the *Pf*START1 mutations, the second half of the coding sequence of *pfstart1* (*PF3D7_0104200*), was recodonised without introns to the bias of *Saccharomyces cerevisiae* containing either WT, N309K or N330K mutations, and synthesised as gBlock fragments (Integrated DNA Technologies). The first 819 bp of the 5′ native sequence of *pfstart1* was amplified from gDNA, to form the 5' homology block (5' HB). The 3′ recodonised gBlock fragment comprising of the last 552 bp of either the WT, N309K or N330K *pfstart1* to form the 'recodon' block (primers listed in Table S2). The 5' HB and recodon products were sewn together by overlapping PCR to reconstitute the full coding sequence of *pfstart1*. These blocks (WT/N309K/N330K) were digested with *Bgl*II/*Pst*I, and inserted into the parasite vector p-HA-2A-Neo-glmS [1]. This plasmid contains an in-frame haemagglutinin (HA) epitope for protein identification and a P2A peptide (2A) to skip continuous peptide bond synthesis of a downstream neomycin resistance gene (*NeoR*).

Parasites containing the transfected DNA were selected using 2.5 nM WR99210 (Jacobus). To select for parasites in which single crossover integration had occurred into the *pfstart1* locus, parasites were treated with 400 µg/mL G418 (geniticin, Sigma Aldrich) for 14 days: only parasites that have integrated the construct into their genome expressed the neomycin resistance gene together with the *Pf*START1 protein. Parasite lines were cloned using limiting dilution. These clones were then tested on Western Blot (for correct HA-tagging and protein size), and correct integration and sequence were tested by PCR analysis: PCR on the gDNA was conducted using the primers START_5'UTR.F and START_Seq.R (Table S2).

Molecular biology and transfection of *P. falciparum*: CRISPR method.

The full 5' homology region of *pfstart1* (either WT, N309K or N330K) was amplified from pPfSTART1-HA-2A-Neo-glmS with the same 5' *Bgl*II primer and a new 3' primer that replaced with *Pst*I with *Spe*I and introduced a stop codon. The PCR product was then digested with *Bgl*II and *Spe*I and inserted into similarly digested p1.2 parasite vector[2]. The p1.2 vector had previously been inserted with a homology region amplified from the 3' end of the *pfstart1* gene (3'HB) to form a second homology block for double recombination. The primers used to amplify 3'HB are listed primers listed in Table S2.

60 µg of p1.2-PfSTART1(WT/N309K/N330K) plasmid was linearised with *Kas*I, precipitated in 3 M sodium acetate and ethanol (70% final concentration) overnight at -20°C. The DNA pellet was washed in cold 70% ethanol and resuspended in cytomix[3]. 50 µM of crRNA (Table S2) and 50 µM of tracRNA (Integrated DNA Technologies) were allowed to form a duplex (5 min at 95°C), and Cas9 enzyme (Integrated DNA Technologies) was added, and incubated for 20 min at room temperature[4]. This crRNA-tracRNA-Cas9 mix was added to each DNA preparation and transfected to 3D7 ring parasites using the Bio-Rad Gene Pulser at 310 V and 950 µF.

Parasites containing the transfected DNA were selected using 2.5 nM WR99210 (Jacobus). Parasites lines were cloned using limiting dilution. Correct integration was tested by PCR (using primer START_5'UTR.F and START_Int.R1, Table S2), and sequencing was done using the primers START_3F and START_Int.R2 (Table S2).

Growth inhibition assays. Synchronised ring-stage parasites (0.3-0.6% parasitemia, 2% haematocrit) were grown for 72 h in a serial-dilution of the compound under investigation: MMV006833 (M-833), compound 2, WEHI-991 (W-991), compound 3

and compound 4. Note that M-833 was first purchased from MolPort (MolPort-002-267-916) then made in-house at WEHI: the differences observed in EC_{50}s across experiments are attributed to the different batches of M-833. For some experiments, 0, 0.25 or 2.5 mM of glucosamine (GlcN) was also added. Experiments were set up in technical duplicates or triplicates. Following a freeze-thaw cycle, lactate dehydrogenase (LDH) activity was assessed as an indicator of parasite growth [5]. 30 µL of parasite culture was incubated for 30 min in the dark with 75 µL Malstat mixture (0.1 M Tris pH 9.0, 20 g/L lactic acid, pH 7.5, 0.2% Triton-X100, 0.5 g/L acetylpyridine adenine dinucleotide (APAD, Sigma), 200 µg/mL nitroblue tetrazolium (NBT; Sigma), 1 µg/mL phenozine ethosulfate (PES; Sigma)). Absorbance was then measured at 650 nm, transformed using the formula below, plotted against the logarithm of the concentrations, and a non-linear regression (log(inhibitor) vs. response, variable slope) was calculated using GraphPad Prism to assess the half-inhibitory concentration (EC_{50}).

Growth inhibition % = $(1 - \frac{average(sample) - average(uRBC)}{average(DMSO) - average(uRBC)}) * 100$

The DMSO samples were also used to assess growth of parasite in the presence of 0.25 and 2.5 mM GlcN (normalised by DMSO in 0 mM GlcN).

Egress, invasion & recovery assay. To measure egress, invasion and follow up recovery in the presence of different compounds, we adapted the method developed in[6], using Hyp1-Nluc parasites[7]. These parasites episomally express the exported PEXEL protein Hyp1 (PF3D7_0113300) tagged with a NanoLuc luciferase (NLuc) and were selected with 2.5 nM WR99210 (Jacobus). Highly synchronised schizonts were obtained using ML10 (LifeArc) and sorbitol synchronisation, and Percoll-purified (the culture was gently layered over 67% Percoll (Cytiva) in RPMI, and centrifuged for 15 min at 1500 g and the schizont layer was collected and washed). In a 96-well plate,

cultures were set up in 4 replicates (3 replicates for the Nluc readout, and one replicate for Giemsa smears) with 1-2% parasitemia, 2% haematocrit, with the following treatment: 2 µM M-883, 60 nM W-991, 0.02% DMSO (Sigma), 25 nM ML10 [8] (LifeArc) 100 µg/ml heparin (Sigma) and 10 µM E64 (Sigma). The schizonts were allowed to egress and invade for 4 h at 37°C. A "background" plate without treatment was left in the fridge during this time. After 4 h, cultures were sorbitol-treated to eliminate schizonts that have not egressed. Smears were made every day for the following 3 days, and 5 µL of the resuspended cultures were taken to measure Nluc activity (to assess parasite's presence). To measure Nluc activity, 5 µL culture were added to 45 µL NanoGlo reagent (1x Promega lysis buffer and NanoGlo reagent; Promega). The average luminescence of the background cultures was subtracted to the average luminescence of each sample.

Parasite reduction ratio assay. This was performed as previously described [9,10]. Briefly, asynchronous ring-stage parasites at 0.5% parasitemia and 2% haematocrit were exposed to compounds at 10 x EC_{50} of growth for five days with daily drug renewal. After each 24 h, a well from each treatment group was taken and washed three times in complete RPMI and final volume adjusted to 2% haematocrit. The washed culture was then aliquoted into a 96 well U-bottom plate and a 1:3 serial dilution into fresh uninfected RBCs was performed with four technical replicates for each time point. Plates were maintained in normal culturing conditions: media was replaced, and fresh RBCs given 1-2 times a week for three weeks, after which parasite growth was measured via lactate dehydrogenase assays. The number of viable parasites was calculated using the formula $x^{n-1}+1$, where x is the dilution factor and n is the number of wells that contain parasite growth. The viable parasite value was then expressed in log units and parasite reduction ratio was calculated as a decrease in

viable parasites over 48 h. The 99.9% parasite clearance rate was determined using a log-linear regression in GraphPad Prism.

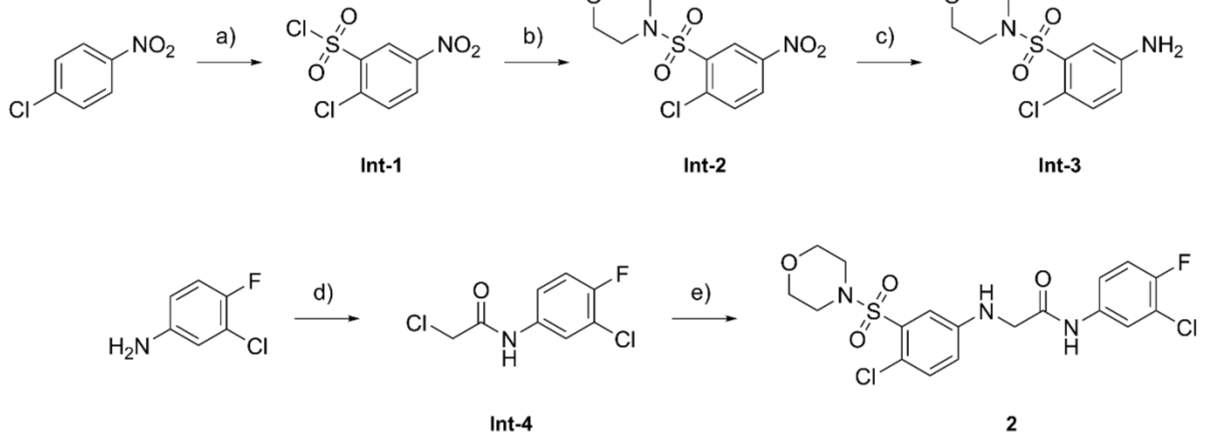

Scheme S1. Synthetic route to generate **2**. *Reagents and conditions:* (a) chlorosulfonic acid, 120 ℃; (b) morpholine, DCM, 0 °C; (c) H_2O: Zn, saturated NH_4Cl (1:1), 40 °C; (d) 2-chloroacetyl chloride, TEA, DCM, 20 °C; (e) **Int-3**, KI, DMF, 45 ℃.

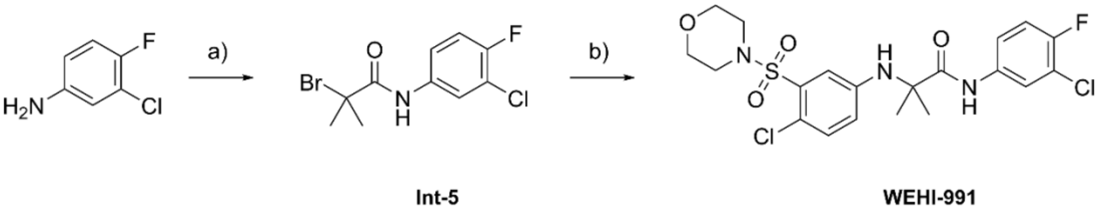

Scheme S2. Synthetic route to generate **WEHI-991**. *Reagents and conditions:* (a) 2-bromoisobutyryl bromide, TEA, DCM, 20 °C; (b) **Int-3**, NaH, THF, 40 °C.

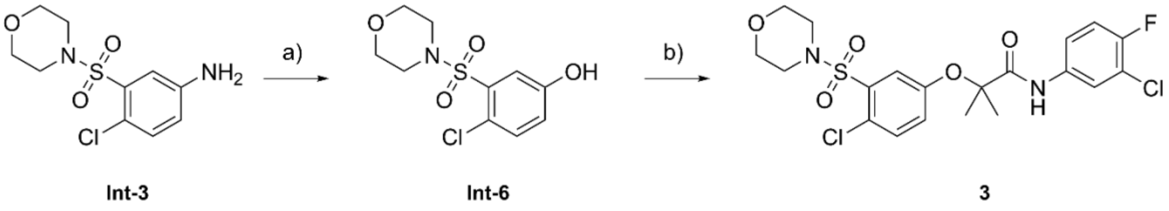

Scheme S3. Synthetic route to generate **3**. *Reagents and conditions:* (a) sodium nitrite, H_2SO_4, H_2O, 0 ℃→65 ℃→100 ℃; (b) **Int-3**, NaH, THF, 40 °C.

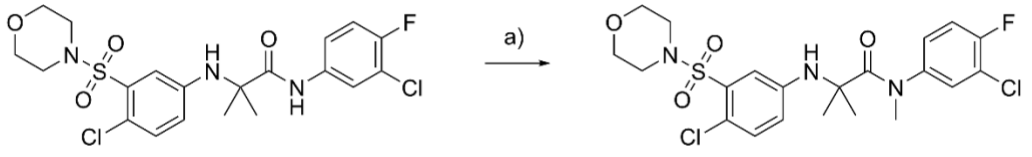

WEHI-991 **4**

Scheme S4. Synthetic route to generate **4**. *Reagents and conditions:* (a) MeI, NaH, THF, 20 °C.

General Chemistry Methods. NMR spectra were recorded on a Bruker Ascend™ 300. Chemical shifts are reported in ppm on the δ scale and referenced to the appropriate solvent peak. Acetone-d_6, and $CDCl_3$ contain H_2O. Chromatography was performed with silica gel 60 (particle size 0.040-0.063 μm) using an automated CombiFlash Rf Purification System. LCMS were recorded on an Agilent LCMS system comprised of an Agilent G6120B Mass Detector, 1260 Infinity G1312B Binary pump, 1260 Infinity G1367E HiPALS autosampler and 1260 Infinity G4212B Diode Array Detector (Method B). Conditions for LCMS Method A were as follows, column: Luna® Omega 3 μm PS C18 100 Å, LC Column 50 × 2.1 mm at 20 ºC, injection volume 2 μL, gradient: 5-100% B over 3 min (solvent A: H_2O 0.1% formic acid; solvent B: ACN 0.1% formic acid), flow rate: 1.5 mL/min, detection: 100-600 nm, acquisition time: 4.3 min. Conditions for LCMS Method B were as follows, column: Poroshell 120 EC-C18, 2.1 × 30 mm 2.7 Micron at 30 ºC, injection volume 2 μL, gradient: 5-100% B over 3 min (solvent A: H_2O 0.1% formic acid; solvent B: ACN 0.1% formic acid), flow rate: 0.8

mL/min, detection: 254 nm, acquisition time: 4.1 min. Unless otherwise noted, all compounds were found to be >95% pure by this method. HRMS were acquired through The Bio21 Mass Spectrometry and Proteomics Facility using a Thermo Scientific™ nano-LC Q Exactive™ Plus Mass spectrometer.

Chemistry procedures.

Int-1

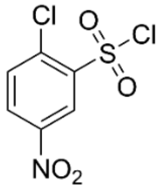

2-Chloro-5-nitro-benzenesulfonyl chloride (**Int-1**). 1-Chloronitrobenzene (1 g, 6.3 mmol) was dissolved in neat chlorosulfonic acid (4.2 mL, 63.4 mmol) and stirred at 120°C for 48 h. The mixture was then slowly pipetted into ice water (20 mL). The resulting precipitate was then collected by vacuum filtration to afford **Int-1** as a brown solid (1.1 g, 68%). ^{1}H NMR (300 MHz, $CDCl_3$): δ 9.02 (d, *J* 2.6 Hz, 1H) 8.52 (dd, *J* 8.7, 2.6 Hz, 1H) 7.89 (d, *J* 8.8 Hz, 1H).

Int-2

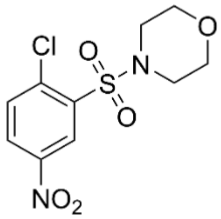

4-(2-Chloro-5-nitro-phenyl)sulfonylmorpholine (**Int-2**). Morpholine (0.405 mL, 4.69 mmol) was added dropwise to a stirred solution of triethylamine (1.09 mL, 7.81 mmol) and **Int-1** (1.0 g, 3.9 mmol) in DCM (20 mL) at 0°C. The reaction was stirred at

this temperature for 3 h. The reaction was then washed with 2M HCl (3 × 20 mL) and the organics dried with anhydrous Na_2SO_4, filtered and concentrated. The crude was then purified by column chromatography eluting with 100% CyHex to 50% EtOAc/CyHex to obtain **Int-2** as a solid (816 mg, 68%). ^{1}H NMR (300 MHz, $CDCl_3$): δ 8.90 (d, *J* 2.7 Hz, 1 H), 8.36 (dd, *J* 8.7, 2.7 Hz, 1 H), 7.76 (d, *J* 8.7 Hz, 1 H), 3.71 - 3.79 (m, 4H), 3.32 - 3.43 (m, 4H). LCMS *m/z* 307.2 [M+1].

Int-3

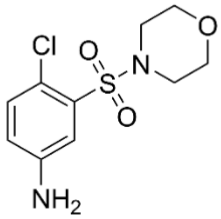

4-Chloro-3-morpholinosulfonyl-aniline (**Int-3**). To a solution of **Int-2** (816 mg, 2.66 mmol) in EtOH (5 mL) and saturated NH_4Cl (5 mL) added zinc (1.7 g, 27 mmol). The mixture was then stirred at 40°C for 3 h. The mixture was then filtered through diatomaceous earth which was washed with EtOH (3 × 10 mL). The filtrate was concentrated, and the resulting residue was taken up into EtOAc (30 mL) and washed successively with saturated $NaHCO_3$ (20 mL), H_2O (20 mL) and brine (20 mL). The organic layer was then dried over anhydrous Na_2SO_4, filtered and concentrated to afford **Int-3** as a solid (480 mg, 65%). ^{1}H NMR (300 MHz, $CDCl_3$): δ 7.35 (d, *J* 2.9 Hz, 1H) 7.32 – 7.27 (m, 1H) 6.79 (dd, *J* 8.5, 2.8 Hz, 1H), 3.71 - 3.78 (m, 4H) 3.26 - 3.36 (m, 4H). LCMS *m/z* 277.2 [M+1].

Int-4

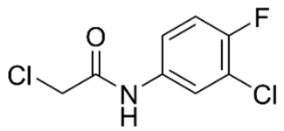

2-Chloro-N-(3-chloro-4-fluoro-phenyl)acetamide (**Int-4**). 2-Chloroacetyl chloride (0.52 mL, 6.6 mmol) was added slowly to a stirred solution of 3-chloro-4-fluoro-aniline (800 mg, 5.5 mmol) and triethylamine (1.5 mL, 11 mmol) in DCM (20 mL) at 20°C. The reaction was then stirred for 16 h at this temperature. The organics were successively washed with 2M HCl (20 ml), saturated $NaHCO_3$ (20 ml) and H_2O (20 ml). The organic layer was dried with Na_2SO_4 and concentrated to afford **Int-4** as a solid (970 mg, 80%). ^1H NMR (300 MHz, $CDCl_3$): δ 8.23 (br s, 1 H), 7.75 (dd, *J* 6.5, 2.6 Hz, 1H), 7.39 (ddd, *J* 9.0, 4.0, 2.7 Hz, 1H), 7.14 (t, *J* 8.7 Hz, 1H), 4.21 (s, 2H). LCMS *m/z* 222.2 [M+1].

Compound 2

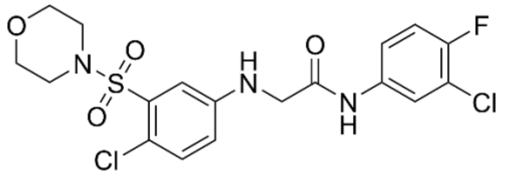

N-(3-Chloro-4-fluoro-phenyl)-2-(4-chloro-3-morpholinosulfonyl-anilino)acetamide (**WEHI-985**). A mixture of **Int-4** (16 mg, 0.072 mmol) in DMF (1 mL) was prepared at 20°C. Potassium iodide (26 mg, 0.16 mmol) was added, and the reaction heated at 45°C for 1 h. The mixture was then cooled to 20°C and **Int-3** (19.9 mg, 0.072 mmol) added in one portion and the mixture heated again to 45°C in a sealed tube for 17 h. The mixture was then diluted with EtOAc (10 mL) and washed with 5% LiCl aq. (3 × 10 mL) and brine (10 mL). The organic layer was dried over anhydrous Na_2SO_4 and concentrated. The crude material was then purified by reverse phase preparatory HPLC using a gradient of 95% H_2O/ACN to 100% ACN to obtain **WEHI-1883985** as a solid (12 mg, 36%). ^1H NMR (300 MHz, $CDCl_3$): δ 8.23 (s, 1 H), 7.71 (dd, *J* 6.5, 2.7 Hz, 1 H), 7.32 - 7.40 (m, 3 H), 7.10 (t, *J* 8.7 Hz, 1 H), 6.76 (dd, *J* 8.7, 2.92 Hz, 1 H), 4.78 (br s, 1 H), 3.96 (d, *J* 5.1 Hz, 2 H), 3.66 - 3.78 (m, 4H), 3.20 - 3.32 (m, 4H). LCMS

m/z 462.2 [M+1]. HRMS acquired: (M + H) 462.0454; $C_{18}H_{18}Cl_2FN_3O_4S$ requires (M + H), 462.0452.

Int-5

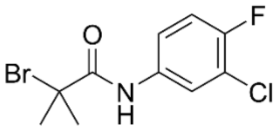

2-Bromo-N-(3-chloro-4-fluoro-phenyl)-2-methyl-propanamide (**Int-5**). 2-Bromoisobutyryl bromide (0.64 mL, 5.2 mmol) was added slowly to a stirred solution of 3-chloro-4-fluoro-aniline (500 mg, 3.4 mmol) and triethylamine (0.96 mL, 6.9 mmol) in DCM (15 mL) at 20°C. The reaction was then stirred for 16 h. The reaction mixture was washed with 2M HCl (20 mL), saturated $NaHCO_3$ (20 mL) and H_2O (20 mL). The organic layer was dried with anhydrous Na_2SO_4 and concentrated to afford **Int-5** as a solid (1.0 g, 99%). ^{1}H NMR (300 MHz, $CDCl_3$): δ 8.44 (br s, 1H), 7.75 (dd, J 6.5, 2.7 Hz, 1H), 7.32 - 7.40 (m, 1H), 7.13 (t, *J* 8.8 Hz, 1H), 2.05 (s, 6H).

WEHI-991

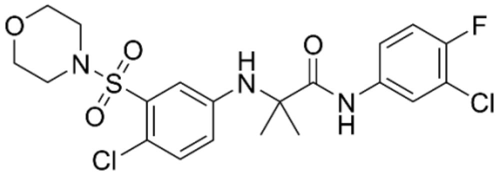

N-(3-Chloro-4-fluoro-phenyl)-2-(4-chloro-3-morpholinosulfonyl-anilino)-2-methyl-propanamide (**WEHI-991**). NaH (60% dispersed in mineral oil, 19.5 mg, 0.488 mmol) was added to a stirred solution of **Int-3** (90 mg, 0.33 mmol) in THF (2 mL). This was stirred for a further 10 min and then **Int-5** (150 mg, 0.52 mmol) was added, and the reaction stirred at 40°C for 16 h. The reaction was then quenched with 5% citric acid (1 mL) and concentrated. The crude was then suspended between EtOAc (10 mL) and washed with sat $NaHCO_3$ (10 mL), and brine (10 mL). The organic layer was then dried

with anhydrous Na_2SO_4, filtered and concentrated. The crude was then purified by column chromatography eluting with 100% DCM to 40% EtOAc/DCM to obtain **WEHI-991** as a solid (72 mg, 45%). ^{1}H NMR (300 MHz, $CDCl_3$): δ 8.66 (s, 1H), 7.71 (dd, *J* 6.5, 2.7 Hz, 1H), 7.29 - 7.38 (m, 3H), 7.08 (t, *J* 8.7 Hz, 1H), 6.64 (dd, *J* 8.7, 2.9 Hz, 1H), 4.35 (s, 1H) 3.66 - 3.74 (m, 4H), 3.21 - 3.29 (m, 4H), 1.60 (s, 6H). LCMS *m/z* 490.4 [M+1]. HRMS acquired: (M + H) 490.0769; $C_{20}H_{22}Cl_2FN_3O_4S$ requires (M + H), 490.0765.

Int-6

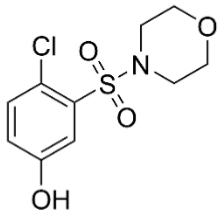

4-Chloro-3-morpholinosulfonyl-phenol (**Int-6**). **Int-3** (310 mg, 1.1 mmol) was dissolved in H_2O (2 mL) and the mixture stirred at 20°C. Concentrated sulfuric acid (0.30 mL, 5.7 mmol) was added drop wise through dropping funnel and the reaction mass was stirred for a period of 15 min. The reaction mixture was then cooled 0°C in an ice bath. Sodium nitrite (117 mg, 1.7 mmol) in H_2O (2 mL) was then added dropwise to the reaction mixture. The resultant mixture was stirred at 0°C for 1 h. In another mL flask, ice-cold water (1 mL) was taken and added concentrated sulfuric acid (1 mL) to it slowly in 10 min. The mixture was warmed to 65°C and the above diazotised mixture was added dropwise. The resulting mixture was stirred at 100°C for 1 h. The reaction mixture was then poured onto ice water (20 mL) under stirring and the resulting mixture was extracted with EtOAc (3 × 15 mL). The combined organics were then washed with H_2O (20 mL) and brine (20 mL), and then dried over anhydrous Na_2SO_4, filtered and concentrated. The crude was then purified by column chromatography eluting with

100% DCM to 50% EtOAc/DCM to obtain **Int-6** as an oil (125 mg, 40%). ^{1}H NMR (300 MHz, $CDCl_3$): δ 7.54 (d, *J* 3.1 Hz, 1 H), 7.40 (d, *J* 8.7 Hz, 1H), 7.00 (dd, *J* 8.7, 2.9 Hz, 1H), 3.71 - 3.78 (m, 4H), 3.28 - 3.33 (m, 4H). LCMS *m/z* 278.2 [M+1].

Compound 3

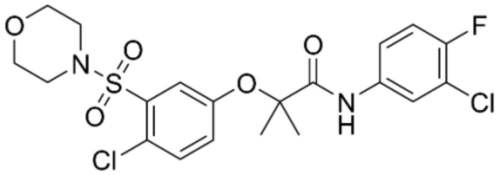

N-(3-Chloro-4-fluoro-phenyl)-2-(4-chloro-3-morpholinosulfonyl-phenoxy)-2-methyl-propanamide (**3**). The procedure used for **WEHI-991** was followed using NaH (60% dispersed in mineral oil, 8.6 mg, 0.22 mmol), **Int-6** (30 mg, 0.11 mmol) and **Int-5** (64 mg, 0.22 mmol) to obtain crude product in 90% purity (39 mg, 66%). 20 mg of this crude was then purified by reverse phase preparatory HPLC using a gradient of 95% H_2O/ACN to 100% ACN to obtain **3** as a solid (9.2 mg, 18%). ^{1}H NMR (300 MHz, $CDCl_3$): δ 8.37 (s, 1H), 7.78 (dd, *J* 6.5, 2.7 Hz, 1H), 7.69 (d, *J* 2.9 Hz, 1H), 7.48 (d, *J* 8.7 Hz, 1H), 7.36 - 7.43 (m, 1H), 7.07 - 7.17 (m, 2H), 3.66 - 3.79 (m, 4H), 3.23 - 3.36 (m, 4H), 1.61 (s, 6H). LCMS *m/z* 491.2 [M+1].

Compound 4

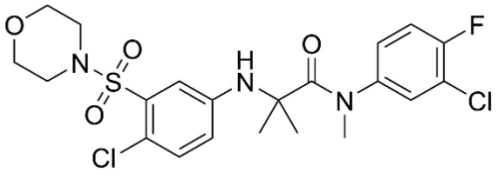

N-(3-Chloro-4-fluoro-phenyl)-2-(4-chloro-3-morpholinosulfonyl-anilino)-N,2-dimethyl-propanamide (**4**). NaH (60% dispersed in mineral oil, 1.3 mg, 0.032 mmol) was added to a stirred solution of **WEHI-991** (7.8 mg, 0.016 mmol) in THF (1 mL) at 20°C and

stirred for 10 min. Iodomethane (5 µL, 0.080 mmol) was then added and the reaction stirred for 16 h at this temperature. The reaction was then quenched with 5% citric acid (1 mL) and concentrated. The crude was then suspended between EtOAc (10 ml) and washed with saturated $NaHCO_3$ (10 mL and brine (10 mL), and then dried with anhydrous Na_2SO_4, filtered and concentrated. The crude material was then purified by reverse phase preparatory HPLC using a gradient of 95% H_2O/ACN to 100% ACN to obtain **4** as a solid (1.8 mg, 22%). ^{1}H NMR (300 MHz, $CDCl_3$): δ 7.31 - 7.36 (m, 1H), 6.94 - 7.09 (m, 3H), 6.80 - 6.90 (m, 1H), 6.52 - 6.62 (m, 1H), 3.73 - 3.78 (m, 4H), 3.21 - 3.32 (m, 7H), 1.55 (s, 6H). LCMS *m/z* 504.2 [M+1].

Gametocyte culturing and standard membrane feeding assay (Walter and Eliza Hall Institute). NF54 *P. falciparum* parasites (Walter Reed Army Institute of Research, USA), were synchronized and maintained as described above at 5-10% parasitemia at 4% hematocrit (WEHI ethics number HREC86/17). Once parasitemia reached 8-10% rings, parasites were diluted to 0.65% parasitemia and supplied with gametocyte media as outlined for asexual stage parasites and the substitution of Albumax for 10% Heat-inactivated human serum (Australian Red Cross Bloodbank) and no gentamycin. Mature sexual stage gametocytes were induced by allowing continuous growth cultures without the addition of fresh human RBCs to crash asexual stages and media was replaced daily [11]. W-991 (1 nM, 50 nM or 100 nM) or WM382 [12] (50 nM) was added on day 13-17 with percentage of Stage V gametocytemia evaluated on day 17. Stage V gametocytes were then centrifuged at 13,000 g for 1 min to remove gametocyte media, 0.2 % of stage V gametocytes were resuspended with the mixture of fresh uninfected RBCs and heat-inactivated human serum at 2:3 ratio.

Anopheles stephensi mosquitoes were reared and maintained in the WEHI insectary and were fed a bloodmeal as stated in [12]. On day 7 after feeding, infected mosquitoes

were aspirated into a cup and anesthetized by cooling to 4ºC until immobilized and kept on ice for the dissection. The mosquitoes were then dissected as previously described [12] and collected midguts were maintained in 1X PBS (Gibco) until they were stained with mercurochrome for 20 mins. Oocysts were then quantified using light microscopy.

Gametocyte culturing and standard membrane feeding assay (London School of Hygiene and Tropical Medicine). *P. falciparum* NF54 gametocytes were prepared for blood-feeding to mosquitoes according to the method described by [13], with some slight modifications. Briefly, gametocyte cultures seeded at 2% overall parasitaemia and 4% haematocrit were maintained at 37° C for 17 days with daily media changes. Gametocyte functional viability was assessed by ex-flagellation assay at day 14 post induction, observed by light microscopy. *An. stephensi* (SD500 strain) were reared as described previously [14].

Gametocyte aliquots from a single NF54 culture, produced as above, were incubated with WEHI-991 at 1nM, 50nM or 100nM, 1mM methylene blue (MB; full-block control) or culture medium only (no-drug control) from day 14 until day 17 when SMFA was carried out. Fresh drug-containing media was provided each day during this exposure. Pots of 70-80 two- to five-day old female *An. stephensi* mosquitoes were allowed to feed on 500 µL of the respective culture+drug mixture, presented to each pot in a pre-heated 3D-printed water channel membrane feeder [15], until fully fed. Mosquitoes were placed in an incubator at standard conditions and midguts dissected in 0.25% mercurochrome stain for oocyst counts by light microscopy 7-8 days post-feed.

***In vitro* liver invasion assays.** This was performed essentially as described[16] with the following variations. *In vitro* human liver HCO4 cells (ATCC) were seeded at 1 × 10^5 cells/mL, grown for 24 h and then infected for 2 h with 30,000 *Pb* ANKA mCherry

expressing sporozoites. Following fixation, an IFA with anti-CSP antibody (1:500) followed by anti-mouse Alexafluor 488 (1:1,000; Invitrogen, Carlsbad, CA, USA) labels uninvaded parasites. Fluorescence signal was assessed using an Olympus CKX41 epifluorescence microscope.

Dual gamete formation assays. The compounds were tested in the *P. falciparum* Dual Gamete Formation Assay (PfDGFA)[13]. Briefly, mature stage V gametocytes were exposed to compounds for 48 h at 37°C in 384 well plates in gametocyte culture medium (RPMI 1640 supplemented with 25 mM HEPES, 50 µg/mL hypoxanthine, 4.8 g/L $NaHCO_3$, 2 mM L-glutamine, 5% pooled type AB serum, 0.5% Albumax II (Gibco)) under a 1% O_2, 3% CO_2, 96% N_2 environment. Gametogenesis was then triggered by the addition of 10 µL ookinete medium (gametocyte culture medium supplemented with 100 µM xanthurenic acid and 0.27 µg/mL Cy3-labelled anti-Pfs25 antibody) to each well at room temperature. Plates were then cooled on a metal block at 4°C for four min to ensure even cooling and then stabilised for a further 4 min at 28°C. At 20 min post-induction, male gametogenesis was recorded in each well by automated brightfield microscopy using a x4 objective lens and 1.5x magnifier (x6 effective magnification). Afterward, plates were incubated in the dark at room temperature for 24 h and then female gametogenesis recorded in each well by automated fluorescence microscopy (anti-Pfs25-positive cells). All experiments were performed in quadruplicate with DMSO and cabamiquine (DDD107498)[17] as negative and positive controls respectively. All data was evaluated in comparison to the positive and negative controls to calculate percentage inhibition of male and female gametocytes, and dose response analysis and IC_{50} calculation performed using GraphPad Prism.

Carbonate extraction. This was conducted as previously described [18]. 3D7 schizonts were lysed in 0.1% saponin as described above. The pellet was subjected to five

freeze-thaw cycles in 20 x pellet volume of PBS+PI (PBS with 1x Complete Protease cocktail tablet (Sigma-Aldrich)) (freeze 1 min in a dry ice-ethanol bath; thaw 2 min at 37°C). The ‘PBS soluble fraction’ was recovered after a 30 min centrifugation step (17,000 g at 4°C). After a wash in PBS+PI, the pellet was incubated 30 min in 0.1 M Na_2CO_3 (pH 11) at room temperature. The ‘carbonate soluble fraction’ was recovered after another 30 min centrifugation step, the ‘insoluble fraction’ pellet was washed and resuspended in non-reducing SDS-protein sample buffer (NRSB) to a final volume equivalent to the ‘PBS soluble’ and ‘carbonate soluble’ fractions (also complemented with NRSB). Samples were boiled 10 min at 80°C and run on a Western Blot as described previously.

Proteinase K protection assay. This was conducted as previous described [19]. Percoll-purified schizonts were washed in PBS+PI, and lysed in 10x the pellet volume of equinatoxin [20] (EqtII) (to achieve 100% hemolysis) for 10 min at 37°C. The EqtII supernatant was collected, the pellet was washed 3 times in PBS without protease inhibitors and divided equally between six tubes. The pellets were incubated 20 min on ice with 100 µL PBS -/+ 20 µg/mL proteinase K, 0.005% saponin in PBS -/+ 20 µg/mL proteinase K and 0.005% saponin + 1% TX-100 in PBS -/+ 20 µg/mL proteinase K. Proteinase K activity was stopped with a final concentration of 10% of trichloroacetic acid (TCA, Sigma). Proteins were pelleted 20 min at 17,000 g at 4°C, the supernatant was discarded, and 500 µL cold acetone (Merck) was added and left overnight at -20°C. Samples were centrifuged another 10 min, the supernatant was discarded, the pellets were left to air-dry and were then resuspended in NRSB.

Supplementary References

1 Jonsdottir, T. K. *et al.* Characterisation of complexes formed by parasite proteins exported into the host cell compartment of *Plasmodium falciparum* infected red

blood cells. *Cellular Microbiology* **n/a**, e13332, doi:https://doi.org/10.1111/cmi.13332 (2021).
2 Dans, M. G. *et al.* Sulfonylpiperazine compounds prevent *Plasmodium falciparum* invasion of red blood cells through interference with actin-1/profilin dynamics. *PLOS Biology* **21**, e3002066, doi:10.1371/journal.pbio.3002066 (2023).
3 Wu, Y., Sifri, C. D., Lei, H. H., Su, X. Z. & Wellems, T. E. Transfection of *Plasmodium falciparum* within human red blood cells. *Proc Natl Acad Sci U S A* **92**, 973-977, doi:10.1073/pnas.92.4.973 (1995).
4 McHugh, E. *et al.* Nonsense-mediated decay machinery in *Plasmodium falciparum* is inefficient and non-essential. *mSphere* **8**, e0023323, doi:10.1128/msphere.00233-23 (2023).
5 Makler, M. T. & Hinrichs, D. J. Measurement of the Lactate Dehydrogenase Activity of *Plasmodium falciparum* as an Assessment of Parasitemia. *The American Journal of Tropical Medicine and Hygiene* **48**, 205-210, doi:doi:https://doi.org/10.4269/ajtmh.1993.48.205 (1993).
6 Dans, M. G. *et al.* Screening the Medicines for Malaria Venture Pathogen Box for invasion and egress inhibitors of the blood stage of *Plasmodium falciparum* reveals several inhibitory compounds. *International Journal for Parasitology* **50**, 235-252, doi:https://doi.org/10.1016/j.ijpara.2020.01.002 (2020).
7 Azevedo, M. F. *et al. Plasmodium falciparum* Transfected with Ultra Bright NanoLuc Luciferase Offers High Sensitivity Detection for the Screening of Growth and Cellular Trafficking Inhibitors. *PLoS ONE* **9**, e112571, doi:10.1371/journal.pone.0112571 (2014).
8 Baker, D. A. *et al.* Cyclic nucleotide signalling in malaria parasites. *Open Biol* **7**, 170213, doi:10.1098/rsob.170213 (2017).
9 Sanz, L. M. *et al. P. falciparum* in vitro killing rates allow to discriminate between different antimalarial mode-of-action. *PloS one* **7**, e30949-e30949, doi:10.1371/journal.pone.0030949 (2012).
10 Walz, A. *et al.* The Parasite Reduction Ratio (PRR) Assay Version 2: Standardized Assessment of *Plasmodium falciparum* Viability after Antimalarial Treatment In Vitro. *Pharmaceuticals* **16,** 163 (2023).
11 Vaughan, A. M. *et al.* Complete *Plasmodium falciparum* liver-stage development in liver-chimeric mice. *J Clin Invest* **122**, 3618-3628, doi:10.1172/jci62684 (2012).
12 Favuzza, P. *et al.* Dual Plasmepsin-Targeting Antimalarial Agents Disrupt Multiple Stages of the Malaria Parasite Life Cycle. *Cell Host Microbe* **27**, 642-658.e612, doi:10.1016/j.chom.2020.02.005 (2020).
13 Delves, M. J. *et al.* Routine in vitro culture of *P. falciparum* gametocytes to evaluate novel transmission-blocking interventions. *Nat Protoc* **11**, 1668-1680, doi:10.1038/nprot.2016.096 (2016).
14 Kristan, M., Thorburn, S. G., Hafalla, J. C., Sutherland, C. J. & Oguike, M. C. Mosquito and human hepatocyte infections with *Plasmodium ovale curtisi* and *Plasmodium ovale wallikeri. Trans R Soc Trop Med Hyg* **113**, 617-622, doi:10.1093/trstmh/trz048 (2019).
15 Witmer, K. *et al.* An inexpensive open source 3D-printed membrane feeder for human malaria transmission studies. *Malar J* **17**, 282, doi:10.1186/s12936-018-2436-9 (2018).

16 Rathnapala, U. L., Goodman, C. D. & McFadden, G. I. A novel genetic technique in *Plasmodium berghei* allows liver stage analysis of genes required for mosquito stage development and demonstrates that de novo heme synthesis is essential for liver stage development in the malaria parasite. *PLoS Pathog* **13**, e1006396, doi:10.1371/journal.ppat.1006396 (2017).
17 Baragana, B. *et al.* A novel multiple-stage antimalarial agent that inhibits protein synthesis. *Nature* **522**, 315-320, doi:10.1038/nature14451 (2015).
18 Bullen, H. E. *et al.* The *Plasmodium falciparum* parasitophorous vacuole protein P113 interacts with the parasite protein export machinery and maintains normal vacuole architecture. *Molecular Microbiology* **117**, 1245-1262, doi:https://doi.org/10.1111/mmi.14904 (2022).
19 Jonsdottir, T. K. *et al.* PTEX helps efficiently traffic haemoglobinases to the food vacuole in *Plasmodium falciparum*. *PLOS Pathogens* **19**, e1011006, doi:10.1371/journal.ppat.1011006 (2023).
20 Jackson, K. E. *et al.* Selective permeabilization of the host cell membrane of *Plasmodium falciparum*-infected red blood cells with streptolysin O and equinatoxin II. *Biochem J* **403**, 167-175, doi:10.1042/BJ20061725 (2007).
