# Supplementary material for: Aryl amino acetamides prevent Plasmodium falciparum ring development via targeting the lipid-transfer protein PfSTART1
Source: Nat Commun. 2024 Jun 18;15:5219. doi: 10.1038/s41467-024-49491-8 (PMC11189555; doi:10.1038/s41467-024-49491-8)
Supplement: Supplementary file 3 — Description of Additional Supplementary Files [file 41467_2024_49491_MOESM3_ESM.pdf]

Description of Additional Supplementary Files

File Name: Supplementary Data 1
Description: M-833- resistant genomes pooled variant summary. Table depicts genes in which non-synonymous SNPs were identified that passed quality filtration and were detected in at least one resistant clone. Highlighted rows indicate putative targets.

File Name: Supplementary Movie 1
Description: RBC invasion with DMSO control. Merozoites are stained in cyan (with Mitotracker Deep Red CMXRos) and the RBC membrane is stained in magenta (with Di-4-ANEPPDHQ)

File Name: Supplementary Movie 2
Description: RBC invasion with 60 nM W991 control. Merozoites are stained in cyan (with Mitotracker Deep Red CMXRos) and the RBC membrane is stained in magenta (with Di-4-ANEPPDHQ)
