# Supplementary material for: Aryl amino acetamides prevent Plasmodium falciparum ring development via targeting the lipid-transfer protein PfSTART1
Source: Nat Commun. 2024 Jun 18;15:5219. doi: 10.1038/s41467-024-49491-8 (PMC11189555; doi:10.1038/s41467-024-49491-8)
Supplement: Supplementary file 7 — Reporting Summary [file 41467_2024_49491_MOESM7_ESM.pdf]

nature portfolio

Corresponding author(s): Madeline Dans and Paul Gilson

Last updated by author(s): May 15, 2024

Reporting Summary

Nature Portfolio wishes to improve the reproducibility of the work that we publish. This form provides structure for consistency and transparency in reporting. For further information on Nature Portfolio policies, see our Editorial Policies and the Editorial Policy Checklist.

Statistics

For all statistical analyses, confirm that the following items are present in the figure legend, table legend, main text, or Methods section.

n/a	Confirmed	
☐	☒	The exact sample size (*n*) for each experimental group/condition, given as a discrete number and unit of measurement
☐	☒	A statement on whether measurements were taken from distinct samples or whether the same sample was measured repeatedly
☐	☒	The statistical test(s) used AND whether they are one- or two-sided<br>*Only common tests should be described solely by name; describe more complex techniques in the Methods section.*
☒	☐	A description of all covariates tested
☐	☒	A description of any assumptions or corrections, such as tests of normality and adjustment for multiple comparisons
☐	☒	A full description of the statistical parameters including central tendency (e.g. means) or other basic estimates (e.g. regression coefficient) AND variation (e.g. standard deviation) or associated estimates of uncertainty (e.g. confidence intervals)
☐	☒	For null hypothesis testing, the test statistic (e.g. *F*, *t*, *r*) with confidence intervals, effect sizes, degrees of freedom and *P* value noted<br>*Give P values as exact values whenever suitable.*
☒	☐	For Bayesian analysis, information on the choice of priors and Markov chain Monte Carlo settings
☒	☐	For hierarchical and complex designs, identification of the appropriate level for tests and full reporting of outcomes
☒	☐	Estimates of effect sizes (e.g. Cohen's *d*, Pearson's *r*), indicating how they were calculated

Our web collection on statistics for biologists contains articles on many of the points above.

Software and code

Policy information about availability of computer code

Data collection	Whole genome sequencing: MinION platform with MIN106D Flow cells and MinIT (Software 18.09.1), Porechop (V0.2.3_seqan2.1.1) used for demultiplexing and adaptor trimming. Growth inhibition assays: Multiskan Go plate reader (Thermo Scientific), using Skan IT software 3.2. Egress and parasite reduction ratio assays: CLARIOstar luminometer (BMG Labtech). Stage arrest assay: Attune Flow Cytometer (ThermoFisher Scientific) with images acquired by Nikon Eclipse E600 microscope. Lattice light sheet imaging: Zeiss Lattice Lightsheet 7 with images processed by Zeiss Zen Blue 3.4 software. Isothermal Calorimetry: MicroCal PEAQ-ITC calorimeter. Western Blotting: ChemiDoc Imaging System (Biorad) (Solvent profiling experiments) or Odyssey imaging system (all remaining parasite protein experiments). Mass-spectrometry: Orbitrap Eclipse Tribrid Mass Spectrometer (Thermo Scientific). In vitro liver invasion assays: Olympus CKX41 epifluorescence microscope.
Data analysis	Whole genome sequencing: R statistical software (V3.6.1). Isothermal Calorimetry: PEAQ-ITC software (MicroCal). Mass-spectrometry: R statistical software (V4.2.0). Lattice light sheet imaging: Aivia 10.5.1 software. All remaining data analysis was performed in GraphPad Prism 9.5.0.

For manuscripts utilizing custom algorithms or software that are central to the research but not yet described in published literature, software must be made available to editors and reviewers. We strongly encourage code deposition in a community repository (e.g. GitHub). See the Nature Portfolio guidelines for submitting code & software for further information.

Data

Policy information about availability of data

All manuscripts must include a data availability statement. This statement should provide the following information, where applicable:

- Accession codes, unique identifiers, or web links for publicly available datasets
- A description of any restrictions on data availability
- For clinical datasets or third party data, please ensure that the statement adheres to our policy

Genomic sequencing data is available from European Nucleotide Archive; accession number PRJEB65444. All raw and processed data will be made available upon request.
The protein mass spectrometry data has been submitted to ProteomeXchange with accession number PXD048262. Reviewers can preview the data using Preview Access Link: https://repository.jpostdb.org/preview/1533710161659765488dfc7
Access Code: 8414

Research involving human participants, their data, or biological material

Policy information about studies with human participants or human data. See also policy information about sex, gender (identity/presentation), and sexual orientation and race, ethnicity and racism.

Reporting on sex and gender	N/A
Reporting on race, ethnicity, or other socially relevant groupings	N/A
Population characteristics	N/A
Recruitment	N/A
Ethics oversight	N/A

Note that full information on the approval of the study protocol must also be provided in the manuscript.

Field-specific reporting

Please select the one below that is the best fit for your research. If you are not sure, read the appropriate sections before making your selection.

☒ Life sciences ☐ Behavioural & social sciences ☐ Ecological, evolutionary & environmental sciences

For a reference copy of the document with all sections, see nature.com/documents/nr-reporting-summary-flat.pdf

Life sciences study design

All studies must disclose on these points even when the disclosure is negative.

Sample size	No statistical method was used to predetermine sample sizes but we used similar to previous publications (Dans et al., PMID: 37053271).
Data exclusions	No data was excluded from the analyses.
Replication	To verify reproducibility, at least 3 biological replicates were repeated unless stated in figure legend.
Randomization	Randomization was not done as it was not necessary to randomize the experimental or control groups to avoid bias.
Blinding	Blinding was not as there was no possibly of unconscious bias in the aquisition or interpretation of results.

Reporting for specific materials, systems and methods

We require information from authors about some types of materials, experimental systems and methods used in many studies. Here, indicate whether each material, system or method listed is relevant to your study. If you are not sure if a list item applies to your research, read the appropriate section before selecting a response.

Materials & experimental systems

n/a	Involved in the study
☐	☒ Antibodies
☐	☒ Eukaryotic cell lines
☒	☐ Palaeontology and archaeology
☐	☒ Animals and other organisms
☒	☐ Clinical data
☒	☐ Dual use research of concern
☒	☐ Plants

Methods

n/a	Involved in the study
☒	☐ ChIP-seq
☒	☐ Flow cytometry
☒	☐ MRI-based neuroimaging

Antibodies

Antibodies used

Anti-HA 1:1000
Anti-PfSTART1:1000
Anti-PfEXP2 rabbit 1:2000
Anti-PfEXP2 mouse monoclonal 1:2000
Anti-PfHSP70.1 1:2000
Anti-PfGBP130 1:1000
Anti-PfSERA5 1:1000
Anti- PfHSP101 1:1000
Anti- PfActin-1 1:500
Rabbit-Alexa Fluor Plus 680 1:10000
Mouse-Alexa Fluor Plus 800 1:10000
Rabbit-Alexa Fluor 488 1:2000
Mouse-Alexa Fluor 594 1:2000

Validation

Rabbit-Alexa Fluor Plus 680: https://www.thermofisher.com/order/genome-database/dataSheetPdf?producttype=antibody&productsubtype=antibody_secondary&productId=A32734&version=364
Mouse-Alexa Fluor Plus 800: https://www.thermofisher.com/order/genome-database/dataSheetPdf?producttype=antibody&productsubtype=antibody_secondary&productId=A32730&version=364
Rabbit-Alexa Fluor 488: https://www.thermofisher.com/order/genome-database/dataSheetPdf?producttype=antibody&productsubtype=antibody_secondary&productId=A-11008&version=364
Mouse-Alexa Fluor 594: https://www.thermofisher.com/order/genome-database/dataSheetPdf?producttype=antibody&productsubtype=antibody_secondary&productId=A-11072&version=364
Anti-HA (mouse, Sigma-Aldrich, H3636, Clone HA-7, monoclonal) https://www.sigmaaldrich.com/AU/en/product/sigma/h3663
Anti-PfSTART (rabbit, made in-house WEHI)
Anti-PfEXP2 (mouse, PMID: 19536257)
Anti-PfEXP2 (rabbit, PMID: 19536257)
Anti-PfHSP70.1 (rabbit, PMID: 28732045)
Anti-PfGBP130 (mouse, made in-house WEHI)
Anti-PfSERA5 (rabbit, PMID: 13679369)
Anti- PfHSP101 (rabbit, PMID: 19536257)
Anti- PfActin-1 (mouse, PMID: 22389687)

Eukaryotic cell lines

Policy information about cell lines and Sex and Gender in Research

Cell line source(s)

P. falciparum laboratory wildtype strain 3D7 parasites
P. falciparum NF54 (Walter Reed Army Institute of Research, USA)
P. falciparum START-HA mutants (SLI-method)- see supplementary methods
P. falciparum START mutants (CRISPR method)-see supplementary methods
P. falciparum exported-Nluc (PMID: 25392998)
P. berghei ANKA-mCherry (PMID: 28617870)
In vitro human liver HCO4 cells (ATCC) (PMID: 28617870)

Authentication

Newly made Plasmodium transgenic cell lines were authenticated through western blotting, immunofluoresence microscopy, PCR-based determination of correct genomic integration and genomic sequencing of mutation sites. NC04 cell lines were used in this study.

Mycoplasma contamination

Cell lines were not tested for mycoplasma contamination.

Commonly misidentified lines
(See ICLAC register)

No commonly misidentified cell lines were used in this study.

Animals and other research organisms

Policy information about studies involving animals; ARRIVE guidelines recommended for reporting animal research, and Sex and Gender in Research

Laboratory animals	Laboratory animals- 4- to 5-week-old male Swiss Webster mice and were purchased from the Monash Animal Services (Melbourne, Victoria, Australia) and housed at 22 to 25°C on a 12 h light/dark cycle and 40-70% humidity at the School of Biosciences, The University of Melbourne, Australia.<br>Anopheles stephensi mosquitoes were reared and maintained in the WEHI insectary.
Wild animals	The study did not involve wild animals.
Reporting on sex	Mice were only used to generate Plasmodium sporozoites so sex was not a consideration
Field-collected samples	The study did not involve samples collected from the field
Ethics oversight	All animal experiments were in accordance with the Prevention of Cruelty to Animals Act 1986, the Prevention of Cruelty to Animals Regulations 2008 and National Health and Medical Research Council (2013) Australian code for the care and use of animals for scientific purposes. These experiments were reviewed and permitted by the Melbourne University Animal Ethics Committee (2015123).<br>Mosquito transmission experiments were conducted under WEHI ethics number HREC86/17.

Note that full information on the approval of the study protocol must also be provided in the manuscript.

Plants

Seed stocks	N/A
Novel plant genotypes	N/A
Authentication	N/A
